# *Channa striatus* in inflammatory conditions: A systematic review

**DOI:** 10.3389/fphar.2022.1076143

**Published:** 2022-12-05

**Authors:** Vanessa Lin Lin Lee, Brandon Kar Meng Choo, Anwar Norazit, Suzita Mohd Noor, Mohd Farooq Shaikh

**Affiliations:** ^1^ Neuropharmacology Research Laboratory, Jeffrey Cheah School of Medicine and Health Sciences, Monash University Malaysia, Bandar Sunway, Selangor, Malaysia; ^2^ Department of Biomedical Science, Faculty of Medicine, University of Malaya, Kuala Lumpur, Malaysia

**Keywords:** traditional medicine, *Channa striatus*, inflammation, anti-inflammatory, natural product

## Abstract

*Channa striatus* (CS), or snakehead murrel, is an obligate air-breathing freshwater fish. Besides its wound healing properties, CS has also been reported to exhibit anti-inflammatory effects in multiple studies. While there are anti-inflammatory medications such as nonsteroidal anti-inflammatory drugs (NSAIDs), their long-term use is associated with an increased risk of peptic ulcers, acute renal failure, stroke, and myocardial infarction. Thus, it is essential to look at natural methods such as CS extract. While there is an abundant number of investigative studies on the inflammatory properties of CS, the quality of these studies has not been evaluated effectively. Thus, this review aims to summarise, evaluate, and critically appraise currently available literature regarding the anti-inflammatory properties of CS extract. This is done by performing a search using four databases, namely Google Scholar, Embase *via* Elsevier, Scopus, and Web of Science, with the following terms: *Channa striatus* AND inflammation. From our review, CS has been experimentally shown to positively affect inflammatory conditions such as gastric ulcers, dermatitis, osteoarthritis, and allergic rhinitis. Beneficial effects were also found on inflammation in the presence of tuberculosis and in situations that involve inflammation, such as wound healing. While CS clearly has potential for treating inflammatory conditions, much work needs to be done on identifying and isolating the active constituents before exact mechanisms of action can be worked out to develop future anti-inflammatory medications.

## Introduction

**FIGURE 1:**
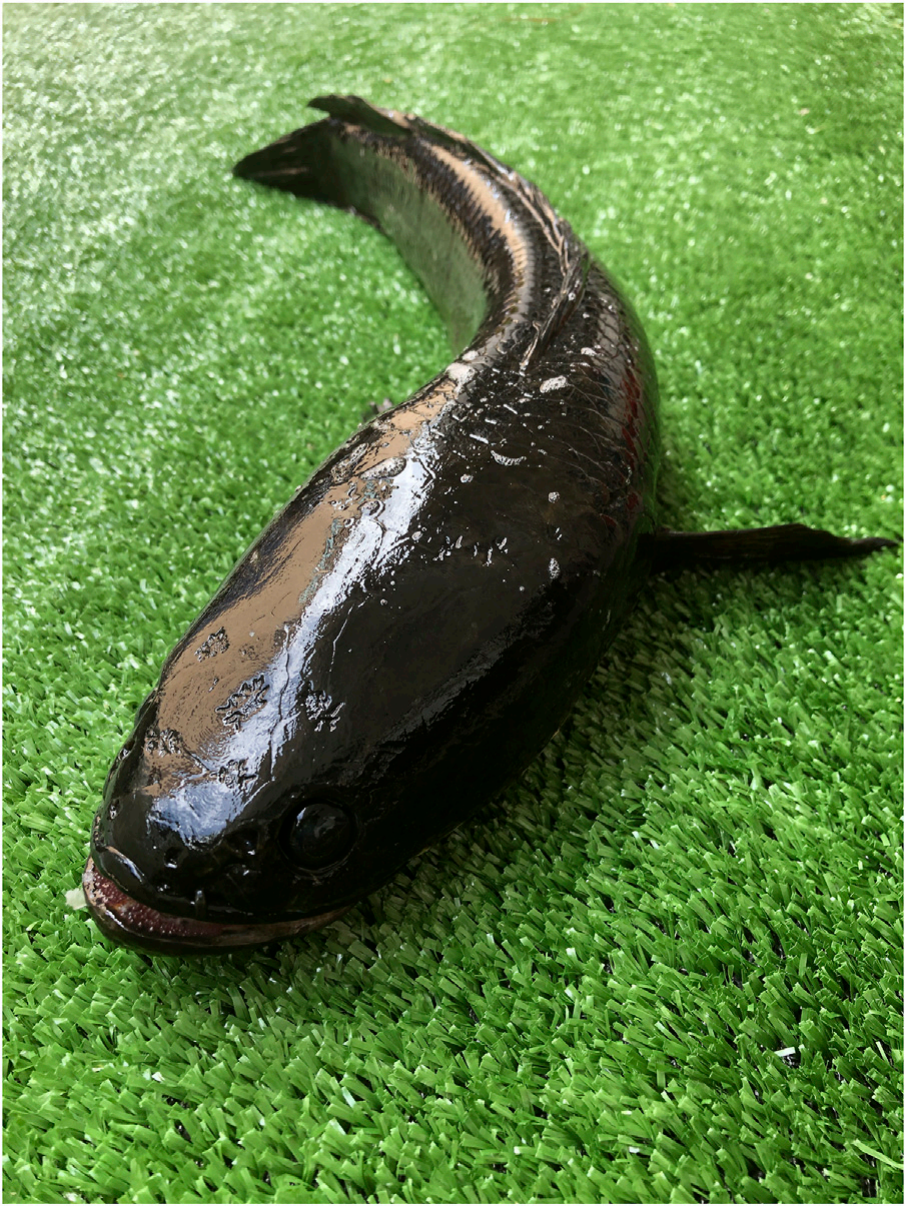
A picture of *Channa striatus*.

*Channa striatus* (CS), or snakehead murrel, is an obligate air-breathing freshwater fish that inhabits all types of water bodies from small ditches to rice fields, rivers, and lakes (Figure 1). It can be found across tropical and subtropical Asian countries from Pakistan and India to Southeast Asia and Southern China (Hossain et al, 2008; Shafri and Abdul Manan, 2012). It belongs to the Channidae family, which has been around since 50 million years ago, with an origin purportedly from the ancient Himalayan Valley (Madeleine, 2004).

Besides its wound healing properties, CS has also been reported to exhibit anti-inflammatory, anti-nociceptive, anti-microbial, and wound healing effects in multiple pre-clinical studies (Jais, 2007; Jais, 2007; Raju et al., 2020; Zakaria, Mat). These pharmacological properties may be contributed by the fact that CS extract contains high levels of major amino acids such as glycine, alanine, lysine, aspartic acid, and proline, as well as fatty acids such as docosahexaenoic acid (DHA), palmitic acid, oleic acid, stearic acid and arachidonic acid (Shafri and Abdul Manan, 2012; Zakaria et al., 2007; Zuraini et al., 2006). Glycine makes up human skin collagen and can also be a polypeptide involved in wound healing together with other amino acids such as alanine, leucine, and phenylalanine, all of which are found in CS extract (Zakaria et al., 2007). Most of these are found in the fillet, but some are found in CS fish roe as well. Roe protein concentrates prepared from CS were also reported to have high antioxidant activity, thus warranting its selection as a functional ingredient in preparing specialty food products (Galla et al, 2012).

Traditionally, CS or more locally known as ‘Haruan' fish, is encouraged to be consumed as part of a post-partum diet, especially for womenwho underwent a cesarean section, because it is believed that CS enhances wound healing as well as improves pain and trauma (Poh et al, 2005; Haniffa et al, 2014). In Malaysia, CS is commonly prepared by frying, roasting, or cooking with curry among the Malay community. Broth or fish tonic made from CS extract are also quite popular as an “energy-restoring” diet (Shafri and Abdul Manan, 2012).

Inflammation is a tissue response to injury caused by hazardous chemicals, physical trauma, or pathological threats (Suhendi et al, 2019). Inflammation is a cascade of events promoted by inflammatory mediators such as histamine, serotonin, kinins, prostaglandins, and interferons (Larsen and Henson, 1983; Weissmann, 2013). Chronic inflammation is universally associated with various diseases such as obesity, cancer, cardiovascular, and neurological disorders (Okin and Medzhitov, 2012). The prevalence of such diseases varies, with symptomatic osteoarthritis occurring in around 10%–13% of Americans older than 60 (Zhang and Jordan, 2010) and allergic rhinitis afflicting an average of 23% of adults in several European countries (Bauchau and Durham, 2004). While there are anti-inflammatory medications such as nonsteroidal anti-inflammatory drugs (NSAIDs), their long-term use is associated with an increased risk of peptic ulcers, acute renal failure, stroke, and myocardial infarction (Marcum and Hanlon, 2010). Anti-inflammatory drugs also interfere with essential cellular pathways (Ghosh et al., 2016). Combined with the risks associated with their chronic use, they are ineffective for treating chronic inflammatory diseases. Thus, it is essential to look at natural methods such as CS extract. While there is an abundant number of investigative studies on the inflammatory properties of CS, the quality of these studies has not been evaluated effectively. Thus, this review aims to summarise, evaluate, and critically appraise currently available literature regarding the anti-inflammatory properties of CS extract. To our knowledge, this is the first review to evaluate the highly beneficial characteristic of CS extract, therefore establishing this review as a timely topic that may propagate the development of effective future anti-inflammatory interventions for a growing number of debilitating inflammatory diseases, with minimal consequences.

## Materials and methods

### Search strategy

A literature search was performed on *Channa striatus* extract in relation to inflammation for inclusion in this systematic review. Articles published from January 2010 until March 2020 were identified and retrieved using four databases: Google Scholar, Embase *via* Elsevier, Scopus, and Web of Science. The following terms were used for the literature search: *Channa striatus* AND inflammation. The Boolean operator AND was used to connect both search terms on all four databases.

### Study selection and exclusion/inclusion criteria

The search was limited to articles published in the English language only and original research articles investigating *Channa striatus* in the context of inflammation. Duplicated articles from the literature search were excluded from this review. Reviews, abstracts, book chapters, patents, symposiums, oral and poster presentations in conferences were also excluded due to inadequate data for assessment and comparison with other studies. Finally, articles that were irrelevant to the aim of the review, did not investigate *Channa striatus* in relation to inflammation or inflammatory diseases, and had no full text were also excluded.

## Results

**FIGURE 2:**
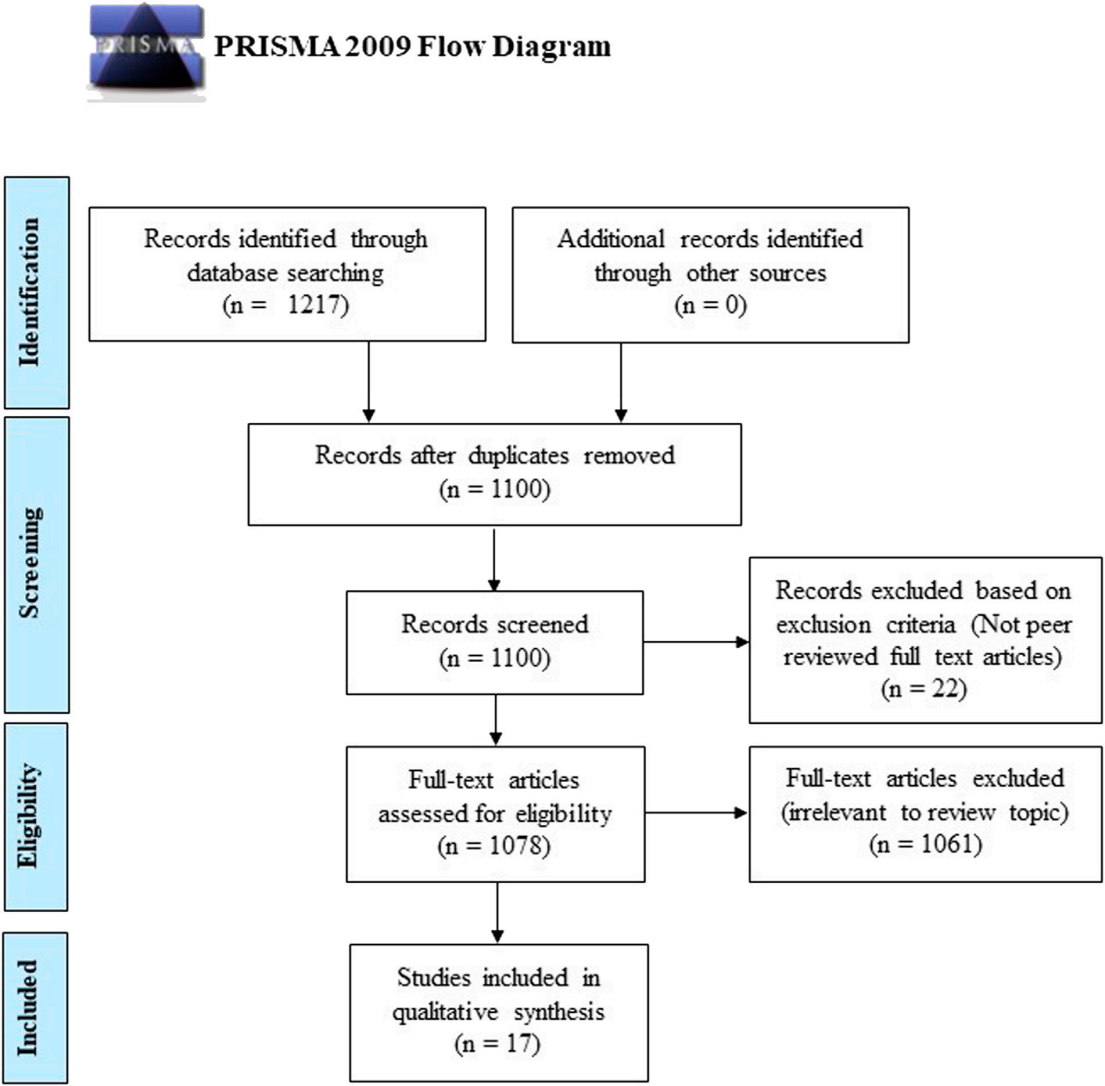
Flow chart showing the article selection and exclusion criteria based on the Preferred Reporting Items for Systematic Reviews and Meta-Analyses (PRISMA) guidelines.

A total of 1,217 articles were identified from the initial database literature search, which was then reduced to 1,100 articles after the removal of duplicates. These 1,100 articles were manually screened according to the aforementioned inclusion and exclusion criteria, and 1,083 articles were excluded as they did not meet the aim of this review or were not full-text original research articles. Thus, based on the PRISMA guidelines, a total of 17 articles were eligible for critical evaluation and appraisal in this study (Figure 2). Among the 16 articles selected, there were seven clinical studies and nine animal studies. These selected articles’ significant findings and characteristics were summarised in Table 1 (animal studies) and Table 2 (clinical studies).

**TABLE 1:** Characteristics and significant findings of the selected pre-clinical studies.

Subjects/Disease model	Inflammatory parameters	Treatment	Significant findings	References
Albino Wistar rats (*n* = N/A) inflammation model	Edema volume	CS powder (150 mg/kg)	The CS extract significantly reduced edema volume in the treatment groups compared to the negative controls	Suhendi et al. (2019)
			The anti-inflammation power of CS extract is comparable to the positive control, diclofenac sodium	
Male Sprague Dawley rats (*n* = 30) gastritis model	Ulcer area and index	Chloroform extract of CS extract (500 mg/kg)- oral	Significant reduction in gastric ulcer formation and ulcer area in a dose-dependent manner, similar to the positive control, 100 mg/kg ranitidine	Azemi et al. (2018)
	Histopathological evaluation		Mild to moderate hemorrhaging and edema to almost normal mucosa architecture in the treated groups compared to severe hemorrhagic erosion, edema, necrosis, and leucocyte infiltration in the vehicle controls	
	Gastric juice parameters		CS extract did not cause significant change to the volume of gastric juice but significantly reduced the total acidity of the gastric juice in a dose-dependent manner	
	Gastric wall mucus			
Male ICR Rats (*n* = 30) dermatitis model	Edema- ear thickness	CS cream 1%, 5%, and 10%	Significant reduction in mouse ear thickness	Isa et al. (2016)
	Histological analysis		Suppression of inflammatory cell infiltration and epidermal hyperplasia was observed	
			All concentrations produced comparable effects to H-cort 1% (positive control)	
	TNFα and GADPH gene expression		A significant reduction in the expression of TNFα levels was observed	
Male Wistar Kiyoto rats (*n* = 48) laparotomy wound healing model	Tensile strength	CS extract (100 mg/100 g)- oral	The tensile strength for the malnourished group treated with oral CS was significantly higher than the untreated control group	Hassan et al. (2020)
	Histological analysis		There was no significant difference in epithelisation count between the two groups	
			CS treatment produced a significantly higher fibroblast count compared with the untreated control group	
			The hydroxyproline measurement for the CS treated group was significantly higher than the untreated control group	
Male Sprague-Dawley rats (*n* = 72), female Sprague-Dawley Rats (*n* = 24), mice and rabbits wound healing model	Irritation test	CS spray	The ability of CS spray to irritate the skin of rabbits was found to be very small	Laila et al. (2011)
	Tensile strength test		Intracutaneous and systemic injection showed that the response from rabbits to CS extracts from four different media were similar to those of the control, indicating that CS spray is safe and non-irritant to the skin of rabbits	
	Burn wound closure		CS spray promotes proliferation of fibroblast cells, increases collagen production and tensile strength	
Male Galur Wistar rats (*n* = 128) wound healing model	Histological analysis	CS extract 25%, 50% and 100% 2.5 ml/250 g	CS extract supplementation increased epithelial thickness at a post-incision area on the rats' buccal mucosa	Tamales et al. (2016)
Adult male ICR rats (*n* = 42) edema model	Ear thickness	CS cream 1%, 5% and 10%	A significant reduction of ear edema in the treatment group was observed	Abedi et al. (2012)
	MPO assay		Edema reduction was as effective as 1% hydrocortisone cream (positive control)	
			The CS creams significantly reduced myeloperoxidase concentration	
Adult male New Zealand white rabbits (*n* = 33) osteoarthritis model	Histological grading	CS extract (51.4 mg/kg)- dry spray	A marked reduction was observed in the macroscopic score compared to the control group in all joint compartments	Abdul Kadir et al. (2019)
	COMP, COX-2, and PGE_2_ serum levels		CS treatment reduced the severity of cartilage lesions compared to the control group	
			Histomorphometrically, CS treated groups demonstrated higher cartilage surface area than the control group	
			The serum level of COMP, a cartilage degradation biomarker, was significantly higher in the control group compared to CS and 77.5 mg/kg glucosamine (positive control) groups	
			No significant difference was observed between all the treatment groups in serum COX-2 and PGE2 levels	
PC12 cells	Neurite outgrowth	CS extract (100, 200, 300, and 400 µl)	Notable neurite outgrowth were observed in CS treated groups compared to negative control, but the growth pattern was different from the cells treated with 1 ml dibutyryl cAMP (positive control)	[M. A. M. Shafri et al, (2011)]

CS, *Channa striatu*; H-cort, Hydrocortisone; TNF-α, Tumor Necrosis Factor-alpha; GADPH, Glyceraldehyde 3-phosphate dehydrogenase; MPO, Myeloperoxidase; COMP, Cartilage oligomeric matric protein; COX-2, cyclooxygenase-2; PGE_2_, prostaglandin E.

**TABLE 2:** Characteristics and significant findings of the selected clinical studies.

Type of study	Treatment	Inflammatory markers	Findings	References
Randomized double-blind, placebo-controlled study- Post Lower Segment Caesarean Section (LSCS) pain study. *n* = 60.	500 mg (2 capsules) of freeze-dried CS extract	C-reactive protein (CRP)	No significant difference in inflammatory markers between CS and control group	Shafii et al. (2017)
		Total white cell counts		
		Platelet count		
Randomized, double-blind, two-arm parallel comparative study- post LSCS study. *n* = 73.	500 mg freeze-dried CS extract daily for 6 weeks	Serum IL-6	CS consumption significantly increased the wound healing biomarkers, IL-6, MMP-9, and VEGF compared to the placebo group	Omar et al. (2020)
		Serum vascular endothelial growth factor (VEGF)	Findings from this study suggested that CS extract increased the cytokines and growth factors in response to tissue injury	
		Serum matrix metalloproteinase 9 (MMP-9)		
Randomized, double-blind, two-arm parallel comparative study- post-LSCS pain study. *n* = 76.	500 mg/day freeze-dried CS extract for 6 weeks	NRS for pain analysis	The VAS and PSS scores were significantly elevated in the CS group than in the placebo group	Ab Wahab et al. (2015)
		WES for wound healing	The CS group showed an insignificant decrease in pain score over time than the placebo group	
		VAS for wound appearance	WES scores were higher in the CS group compared with the placebo group	
		PSS for overall patient satisfaction with wound appearance		
Randomized, double-blind, placebo-controlled 3-arm trial- knee osteoarthritis. *n* = 120.	500 mg/day and 1,000 mg/day of CS extract	Pain score	Significant reductions were found in the pain score in the CS 1000 mg (52.5%) and CS 500 mg (49.2%) individuals as compared to the placebo (30.4%) group	Azidah et al. (2017)
		Stiffness score	CS 1000 mg/day (*p* < 0.05) and CS 500 mg/day had significantly higher stiffness and physical score value compared to the placebo group	
		Physical function score	No significant difference in analgesic scores and serum COMP between the treatment groups were detected	
		Analgesic score		
		Serum cartilage oligomeric matrix protein (COMP)		
Randomized, double-blind, placebo-controlled pilot study- pulmonary tuberculosis. *n* = 36.	Antituberculosis drug supplemented with 2 g CS extract capsule, three times per day for 12 weeks	TNF-α	Supplementation of CS capsules significantly decreased TNF-α, IFN-γ, and IL-10 levels compared to the baseline	Paliliewu et al. (2013)
		IFN-γ		
		IL-10		
Randomized, double-blind, placebo-controlled, parallel-group comparative study- allergic rhinitis. *n* = 46.	500 mg/day oral CS extract for 6 weeks	Total nasal symptoms score (TNSS)	There was no significant difference in TNSS, serum eosinophil, and IL-4 in the CS group compared to the placebo group at week 4	Bakar, (2015)
		Serum eosinophil	In patients with moderate and severe symptoms scores, there were significant differences in TNSS, serum eosinophil, and IL-4 levels between the CS and placebo groups	
		Serum IL-4	Supplementation of CS significantly decreased serum eosinophil count, IL-4 levels, and TNSS at week six compared to the baseline	
			There were no side effects of CS treatment reported in this study	
Randomized, double-blind, placebo-controlled trial- allergic rhinitis. *n* = 70.	500 mg/day CS extract for 6 weeks	Nasal symptom score	Significant reduction of blockage and itchiness symptoms in the treatment group compared to subjects of the placebo group	Susibalan et al. (2018)
		Non-nasal symptom score	Insignificant reduction of sneezing symptoms in the CS group compared to the placebo group was observed	
		Serum IgE	Subjects of the CS group had significant improvement of non-nasal symptoms score in terms of eye itchiness, general symptoms, and serum IgE compared to the placebo group subjects	
			There were no serious adverse events reported in this trial. No significant differences in the laboratory parameters (LFT, RP, and FBC) were observed	

CS, *Channa striatus*; TNF-α, Tumor Necrosis Factor-alpha; IFN-γ, Interferon-gamma; IL-10, Interleukin-10; IgE, Immunoglobulin E; LFT, liver function test; RP, renal profile; FBC, full blood Count; NRS, numeric pain rating scale; WES, Wound Evaluation scale; VAS, Visual Analogue scale; PSS, patient satisfaction score.

### Inflammation model

In a study by Suhendi et al. (2019), an inflammation Wistar rat model was induced using carrageenan 2%. Prior to the induction of inflammation, the groups were treated with either CS powder, Nephelium lappaceum fruit peel extract (NPLE), or a combination of both. Diclofenac sodium was also given as a positive control. From the results, it could be concluded that the CS extract affects reducing edema volume. Based on statistical tests, diclofenac sodium, CS Powder (CSP), *Nephelium lappaceum* fruit peel extract (NPLE), and a combination of CSP and NPLE showed significantly different results (*p* < 0.05) compared with the negative controls. Interestingly, the combination treatment consisting of CSP and NPLE extract did not show a stronger anti-inflammatory effect than the single extract treatment. (Suhendi et al., 2019).

However, we did find some discrepancies in the information given by this journal article. It is unclear if the study was done on rats or mice because these two words seemed to be used interchangeably. The authors also mentioned that diabetes was also induced in this study, but it is unclear if the diabetes was induced in the same animals. Thus it was unclear if the inflammation was measured in a diabetic condition or not.

### Tuberculosis

A randomized, placebo-controlled, double-blind pilot study was conducted on pulmonary tuberculosis (TB) patients to study the effects of CS capsules on cytokine conversion in pulmonary TB patients when given in conjunction with standard anti-TB drugs. The levels of the inflammatory cytokines, TNF-α, IFN-γ, and IL-10, were significantly reduced in the CS group compared to the baseline. This was seen in the control group, except for the IL-10 level, which was not significant. The conclusion was that adjunctive supplementation of CS capsules accelerated the beneficial therapeutic effect of TB chemotherapy, possibly by improving cytokine response (Paliliewu et al, 2013).

### Gastritis

The antiulcer profile of CS extract, given orally at the doses of 50, 350, and 500 mg/kg, was assessed using ethanol- and indomethacin-induced gastric ulcer models. The antiulcer mechanisms of CS extract were determined as follows: 1) anti-secretory activity of CS extract measured using the pyloric ligation rat model, and 2) the role of nitric oxide and sulfhydryl compounds in the modulation of CS extract antiulcer activity. It was found that CS extract exerted significant antiulcer activity in both models of gastric ulcer. CS extract did not change the volume and pH of gastric juice but reduced the total acidity of gastric juice at lower doses. The antiulcer activity by CS extract was reversed by N-methylamide (NEM) but not by N^G^-omega-Nitro-L-arginine methyl ester (L-NAME).

Therefore, the antiulcer activity demonstrated by CS extract was modulated *via* its cytoprotective, but not anti-secretory effect, and in the presence of sulfhydryl compounds, but not nitric oxide (NO) (Azemi et al., 2018).

### Dermatitis

Chronic-like dermatitis on the ears was induced in male ICR mice using 12-0-tetradecanoylphorbol 13-acetate (TPA). Treatment with CS cream was carried out by applying 1%, 5%, and 10% cream two times a day for three consecutive days after TPA application. A significant reduction of mouse ear thickness by the CS creams was observed compared to TPA alone group (negative control). All concentrations of CS creams produced comparable effects to hydrocortisone (H-cort), the positive control.

Histological analysis revealed suppression of inflammatory cell infiltration and epidermal hyperplasia, noticeable under ×400 magnification. The reduction of dermal edema can also be observed, which was more visible under ×100 magnification. Gene expression analysis showed that all concentrations of CS cream downregulated the expression of TNF-α, with a significant reduction in a dose-dependent manner (Isa et al., 2016).

In another study by Abedi et al. (2012), the anti-inflammatory activity of CS-based cream on ear thickness and myeloperoxidase activity was studied using croton oil-induced ear edema. The effects were compared to hydrocortisone 1% cream as the positive control. It was found that all percentages of CS cream (1%, 5%, and 10%) significantly inhibited edema at 4 h and 24 h after croton oil application. Myeloperoxidase assays results showed that applications of these three dosages of CS cream blocked the migration of polymorphonuclear leukocytes to the dermis. The effect is as effective as hydrocortisone 1% cream in a dose-dependent manner (Abedi et al., 2012).

### Osteoarthritis

The chondroprotective activity of CS was evaluated in an osteoarthritis (OA) rabbit model. OA was induced by performing anterior cruciate ligament transection in male New Zealand white rabbits. The articular cartilage was evaluated macroscopically and histologically using semiquantitative and quantitative methods. The levels of serum cartilage oligomeric matrix protein (COMP), cyclooxygenase 2 (COX-2) enzyme, and prostaglandin E2 (PGE2) were also determined. Macroscopic analysis revealed that CS administration significantly lowered the severity grade of the total macroscopic score compared to the control and glucosamine (positive control) groups.

The CS group had lower histopathological changes in the three compartments of the joint compared to the glucosamine group, which had lower histological scoring in two compartments only. The cartilage thickness, area, and roughness of both CS and glucosamine groups were superior to the control group. Serum COMP levels were lower in both CS (*p* < 0.05) and glucosamine (*p* < 0.05) groups compared to the control group (Abdul Kadir et al., 2019).

A randomized, double-blind, placebo-controlled 3-arm trial was conducted comparing oral CS extract and placebo among knee OA patients for a 6-month intervention period. Significant reductions in Western Ontario and McMaster University Osteoarthritis Index (WOMAC) stiffness and function scores were achieved. However, no significant differences were found between the groups regarding analgesic scores, serum cartilage oligomeric matrix protein (COMP) levels, and biochemical parameters. In conclusion, CS extract treatment was more effective than placebo in treating the symptoms of knee OA (Azidah et al., 2017).

### Wound healing

The wound healing property of CS extract was studied on laparotomy wound healing in malnourished male Wistar Kyoto rats. The study evaluated the effects of CS on tensile strength, epithelialization, and fibroblastic proliferation. CS extract treatment groups demonstrated better tensile strength and significantly higher epithelial and fibroblast cell counts than placebo control (Hassan et al., 2020). A similar study also found that CS has similar positive effects on tensile strength and epithelial and fibroblast cell counts in malnourished male Wistar Kyoto rats when given orally in tablet form and applied topically as a cream (Pasha et al, 2015).

In a separate study, the wound healing effect of CS extract spray was studied in Sprague Dawley rats. Here, CS extract was formulated in an aerosol system, producing a film for wound dressing. CS spray increased the tensile strength of the incision wound and sped up the wound contraction process. This showed that the CS water spray is effective and safe for application to incision and burn injuries (Laila et al., 2011).

Another study by Tamales et al, (2016) was done to study the effect of CS extract on the reepithelization count in the wound healing process. An incision was made in the buccal mucosa area of Galur Wistar rats, and CS extract was administered orally. It was found that CS extract treatment increased epithelial thickness count compared to the negative control, but the difference was not significant (Tamales et al., 2016).

In a randomized, double-blinded study amongst post-Lower Segment Caesarean Section (LSCS) women, CS extract was found to have significant effects on IL-6, Vascular Endothelial Growth Factor (VEGF), and Matrix metallopeptidase 9 (MMP-9) levels between the CS treatment group and placebo group. These factors are involved in different wound healing phases, which suggests that CS extract has potential wound healing properties (Omar et al, 2020). Ab Wahab et al. (2015) found that CS extract significantly improved visual analog scale (VAS) and patient satisfaction score (PSS) in post-LSCS women in a separate study. Although there was no significant effect on postoperative pain and wound evaluation scale (WES), CS extract produced a marked difference in wound cosmetic appearance (Ab Wahab et al., 2015).

In a separate randomized, double-blinded, placebo-controlled study among post LSCS women, however, there were no significant differences in the inflammatory markers during wound healing between the CS extract group and the placebo group (Shafii et al., 2017).

### Allergic rhinitis

A study conducted by Susibalan et al, (2018) on allergic rhinitis subjects revealed that CS treatment significantly improved nasal blockage, nasal itchiness, eye itchiness, and general symptoms compared to the placebo groups. Serum Immunoglobulin E (IgE) was also significantly lowered in the CS group compared to placebo. There were no significant differences between groups regarding nasal discharge, sneezing, palate itchiness, and smell score. This showed some beneficial role in improving nasal symptoms in allergic rhinitis subjects (Susibalan et al., 2018).

In a study by Bakar (2015), CS extract did not significantly reduce Total Nasal Symptoms Score (TNSS), serum eosinophil, and IL-4 in the CS group compared to the placebo group. However, a significant decrement of these parameters was found within the CS treatment group (Bakar, 2015).

## Discussion

After analyzing the available literature on the use of *Channa striatus* derived treatments in inflammatory conditions, beneficial effects were found for several conditions. These conditions were gastric ulcers, dermatitis, osteoarthritis, and allergic rhinitis. Beneficial effects of CS were also found in wound healing as well as on inflammation in the presence of tuberculosis. All the studies in this review used some form of CS extract rather than working with isolated extract constituents. Nevertheless, the studies in the review pointed out several constituents which could be responsible for the beneficial effect of CS extract. These include albumin (Suhendi et al., 2019), the fatty acids linoleic acid, stearic acid, oleic acid, and N-arachidonylglycine, (Abedi et al., 2012), omega-6 polyunsaturated fatty acids, vitamins A, B, E, and D, and minerals such as calcium, sodium, magnesium, and zinc (Paliliewu et al., 2013), omega-3 fatty acids such as eicosapentaenoic acid and docosahexaenoic acid (Susibalan et al., 2018). Excluding the ubiquitous vitamins and minerals, albumin and fatty acids appear to be leading candidates for the anti-inflammatory action seen in CS extract. The two constituents are related as although albumin has antioxidant properties due to its ability to scavenge free radicals, it is also capable of binding fatty acids (Roche et al, 2008). Thus, not only is albumin anti-inflammatory, but by binding fatty acids and facilitating its transport around the body (van der Vusse, 2009), albumin could be enhancing the anti-inflammatory effects of fatty acids. Omega-3 and 6 polyunsaturated fatty acids (Wall et al, 2010), the omega-9 fatty acid, oleic acid (Santamarina et al., 2021), and stearic acid (Pan et al., 2010) are also are known to be anti-inflammatory. Curiously, CS extract also contains arachidonic acid (Che Ku Daud et al, 2010), a known contributor to inflammation. It is possible that CS extract has a net anti-inflammatory effect overall due to its other constituents, or it could be due to CS extract also containing N-arachidonylglycine (Abdul Kadir et al., 2019), which is derived from arachidonic acid but inhibits inflammation instead (Succar et al, 2007).

Knowing the possible active constituents of CS extract is only half the battle won as the mechanism by which it works is also essential. However, we found that a majority of the studies in the review attributed the positive effects of CS extract on the inflammatory conditions to its anti-inflammatory properties in general without identifying specific mechanisms. One of the rare studies in the review that did postulate a mechanism stated that CS extract has a cytoprotective effect by positively modulating the free radical scavenging action of non-protein sulfhydryl compounds (Azemi et al., 2018). Another study pointed to the inhibition of inflammatory cells due to a downregulation of TNF-α (Isa et al., 2016). Different research hypothesized the mechanism to be a downregulation of not only TNF-α but also IFN-γ and IL-10 (Paliliewu et al., 2013). The final study pointed towards a reduction in IgE levels and the chemokines, IL-5 and IL-8 (Susibalan et al., 2018). While an in-depth discussion regarding the mechanisms of anti-inflammatory action is outside the scope of this review, the previously postulated mechanisms of action point to the involvement of the JAK-STAT signaling pathway due to the participation of IL-10, IFN-γ, and IL -5 (Banerjee et al, 2017). Notably however, IL-10 is an anti-inflammatory cytokine (Iyer & Cheng, 2012), and therefore its apparent downregulation could be due to a lack of need because of the overall anti-inflammatory effect of the CS extract, rather than the extract directly suppressing it.

On the other hand, the involvement of TNF-α and IL-8 suggests an effect on the Nuclear Factor kappa-light-chain-enhancer of activated B cells (NF-κB) and c-Jun N-terminal kinases (JNKs) (Bradley, 2008). In the case of allergic inflammatory responses, the reduction in IgE levels is likely a consequence of a reduction in IL-5 levels, possibly by interfering with its production by T-helper 2 cells. This review also included articles looking into wound healing. While the study on wound healing did not look into the anti-inflammatory effects of CS extract specifically, inflammation is a normal part of wound healing. However, wound healing becomes impaired when the inflammation becomes excessive or persistent (Eming et al, 2007). Thus, the wound healing properties of CS extract could also be related to its ability to dampen the inflammatory response.

## Conclusion and future directions

From our review, CS has been experimentally shown to positively affect inflammatory conditions such as gastric ulcers, dermatitis, osteoarthritis, and allergic rhinitis. Beneficial effects were also found on inflammation in the presence of tuberculosis and in situations that involve inflammation in some capacity, such as wound healing. While we were able to suggest several anti-inflammatory pathways that CS might act upon, the use of CS extract by all the studies in this review rather than isolated constituents somewhat clouded the exact mechanisms of action. CS extract is anti-inflammatory overall, even though it contains arachidonic acid, which is pro-inflammatory. While CS clearly has potential for treating inflammatory conditions, more work needs to be done on identifying and isolating the active constituents before exact mechanisms of action can be worked out. Proirities should lie in studying the active compounds which contributes to the pharmacological activities of CS extract. Future pre-clinical and clinical studies can be done using these compounds instead of the crude extract. The candidates for active constituents pointed out in this review will hopefully help in the search for future anti-inflammatory medications.

## Data Availability

The raw data supporting the conclusion of this article will be made available by the authors, without undue reservation.
